# MicroRNAs as Biomarkers in Colorectal Cancer

**DOI:** 10.3390/cancers9090124

**Published:** 2017-09-13

**Authors:** Takaaki Masuda, Naoki Hayashi, Yosuke Kuroda, Shuhei Ito, Hidetoshi Eguchi, Koshi Mimori

**Affiliations:** Department of Surgery, Kyushu University Beppu Hospital, 4546 Tsurumihara, Beppu 874-0838, Japan; takaaki.masuda.280@m.kyushu-u.ac.jp (T.M.); isaya_hikoan@yahoo.co.jp (N.H.); yohsuke.209@m.kyushu-u.ac.jp (Y.K.); sito@med.kyushu-u.ac.jp (S.I.); heguchi@beppu.kyushu-u.ac.jp (H.E.)

**Keywords:** microRNA, biomarker, colorectal cancer

## Abstract

MicroRNAs (miRs) are small RNAs that repress mRNA translation, resulting in the degradation of mRNAs and regulation of the expression levels of various genes. Recent studies have shown that aberrant miR expression has a functional role in the initiation and progression of various malignancies, including colorectal cancer (CRC), which is one of the leading causes of cancer-related death worldwide. miRs have also been shown to have applications as diagnostic, prognostic, and predictive biomarkers because of their high tissue specificity, stability, and altered expression in tumor development. In this report, we examined the role of miRs as biomarkers in CRC through a review of meta-analyses and large-scale analyses having strong statistical confidence in the study outcomes. We also discuss current issues in the clinical application of these miRs.

## 1. Introduction

The incidence of colorectal cancer (CRC) is increasing worldwide. In Japan, CRC is the leading cause of death in women. Many researchers are actively pursuing molecular biological analyses of the mechanisms involved in the onset and progression of CRC. We now know that genetic and epigenetic abnormalities lead to characteristic gene expression profiles that are strongly linked to clinical outcomes [1,2].

Recent reports have demonstrated the presence of noncoding (nontranslational) RNAs (ncRNAs), which do not carry any genetic information for protein synthesis, in the genome. Many subtypes of ncRNAs have been discovered [3]. MicroRNAs (miRs) are small ncRNAs comprised of 18–25 bases. They bind to the 3′ noncoding region of the target mRNA and inhibit the expression of multiple target genes, thereby regulating various cell functions in the body (e.g., differentiation, cell proliferation, and apoptosis) [4,5]. Currently, over 2500 types of miRs have been identified in humans. Associations with various diseases, infectious diseases, central nervous system disorders, and cardiovascular disease have been reported. The initial evidence implicating miRs in CRC pathogenesis was provided in a study by Michael et al. [6], which indicated that *miR-143* and *miR-145* are downregulated in CRC. Since then, many additional studies have demonstrated the tumor suppressive or oncogenic functions of miRs and have described the applications of miRs in the clinical setting as biomarkers or therapeutic targets for CRC.

In the current report, we examined the role of miRs as biomarkers in CRC through a review of meta-analyses and large-scale analyses having strong statistical confidence in the study outcomes. We also discuss current issues in the clinical application of these miRs.

## 2. Methods

For the literature search using PubMed, we used the following search terms on 9 August 2017: “microRNA or miR” AND “colorectal cancer or colon cancer” AND “biomarker.” We then extracted papers related to miRs as a diagnostic, prognostic, or predictive biomarker with meta-analyses or large-scale analyses (total number of samples >100). We found four papers with meta-analyses of prognostic or diagnostic markers. These papers are described in the Tables. We did not list the papers which are described in these papers with meta-analyses in the Tables.

## 3. Biomarkers for the Early Detection and Prediction of Prognosis in CRC

In order to achieve favorable outcomes, early detection of CRC is critical. To this end, clinicians often use fecal occult blood tests (FOBTs) and colonoscopy together or separately [7,8,9]. FOBTs are performed annually and are noninvasive screening tests that are used to detect blood in stool; these tests have yielded an estimated 24–39% reduction in CRC-related mortality, but are not highly sensitive. Indeed, positive FOBT results lead to frequent colonoscopy screening [9], which is the most common method for detection of CRC. Colonoscopy reduces the risk of CRC by 30–75%; however, an estimated 25% of polyps are not detected during colonoscopy, and the technique is expensive and invasive [9,10,11]. Given these caveats, cheaper, less-invasive, and more quantitative tests would provide an attractive alternative to the current standard screening methods.

Recently, miRs have been highlighted as diagnostic, prognostic, and predictive biomarkers because of the high tissue specificity, stability, and altered expression in tumor development [12,13]. Thus, miR analysis could offer a less-invasive and more cost-effective alternative to supplement existing screening approaches.

### 3.1. miRs in Tumor Tissues

Tumor tissue is the most important source for the identification of CRC-related miRs. miRs are expressed differently in cancer and normal cells and therefore may constitute a reliable diagnostic biomarker. The roles of miRs in CRC were initially identified by Michael et al. [6], who discovered that *miR-143/miR-145* were significantly reduced in CRC [14].

Various miRs, including *miR-101* [15], the *let7* family, *miR-133b*, *miR-126* [16], and *miR-142-3p* [17], have been found to act as tumor suppressors (tumor-suppressive miRs). Some miRs are highly expressed in CRC cells, playing a major role in creating a microenvironment that allows the cancer cells to thrive. These miRs are known as oncomirs (oncogenic miRs). One of the earliest miRs to be identified as an oncomir was *miR-21*, which plays an important role in the initiation, progression, and metastasis of CRC [18]. Recent studies have identified many miRs as prognostic biomarkers in CRC tissues (Table 1).

### 3.2. miRs in Stool

CRC-specific miRs can also be detected in stools. Analysis of stool miRs has attracted much interest in recent years as a potential noninvasive diagnostic tool for early CRC screening. Tumor cells that are shed from CRC tissues can provide valuable genetic and epigenetic information to facilitate tumor detection [64].

Many studies have reported differential expression of miRs in the stools of patients with CRC [65,66,67,68,69,70,71,72,73]. The feasibility of isolating stool RNAs and quantifying mature miRs by reverse transcription polymerase chain reaction (RT-PCR) was initially demonstrated in a study by Ahmed et al., in which seven miRs, including *miR-320*, *miR-126*, *miR-484-5p*, *miR-143*, *miR-145*, *miR-16*, and *miR-125b*, were downregulated in stool collected from patients with CRC when compared with those of healthy controls [65].

A study by Link et al. demonstrated the presence of higher levels of *miR-21* and *miR-106a* in the stool of patients with CRC and colorectal adenoma compared with that in healthy controls by RT-PCR and microarray analysis [68]. Yau et al. found that the expression of *miR-221* and *miR-18a* increased steadily with tumor initiation and progression using an miR expression array [73]. Koga et al. reported that the overall sensitivity and specificity of stool analysis based on upregulation of the *miR-17-92* cluster, *miR-21*, and *miR-135* were 74% and 79%, respectively, suggesting that miR expression analysis in stool samples could be a useful method for CRC screening [72].

Notably, stool *miR-29* was found to be significantly more abundant in patients with rectal cancer than in those with colon cancer, suggesting the feasibility of using differential miR expression patterns as cancer fingerprints [71]. Recent studies have identified many miRs in the stool as diagnostic biomarkers for CRC (Table 2). However, despite the promise of stool miRs for detection of CRC, there are challenges associated with obtaining and handling the samples.

### 3.3. miRs in Serum/Plasma

Cancer cells release miRs into the peripheral blood [92]. Thus, circulating miRs could be detected from serum and plasma; these molecules are currently being exploited aggressively as potential biomarkers for the diagnosis and monitoring of cancer progression or treatment in patients with CRC.

The first report of miRs detected in the serum of patients with CRC was published by Chen et al. in 2008 [93]. They identified 69 miRs in the serum of patients with CRC but not in the serum of healthy controls.

Circulating miRs are present in various forms and have even been found within exosomes. As extracellular vesicles secreted from cells by exocytosis, exosomes are found in most circulating body fluids and contain proteins, mRNAs, and miRs [94]. Notably, exosomal miRs are more stable than other miR forms because they are not degraded by endogenous RNase. Accordingly, exosomal miRs may have potential applications as cancer-specific biomarkers.

Exosomes are key contributors to intercellular communication. Exosomes can carry a number of molecules, such as DNAs, mRNAs, proteins, and miRs, to recipient cells [95]. Hence, exosomes and their cargo transfer specific messages to recipient cells and change the behavior of these cells. Many studies have shown that exosomes released from cancer cells are important players in tumor progression in several diseases, including CRC [95,96]. We previously reported that abundant expression of exosomal *miR-19a* in serum was a prognostic biomarker for recurrence in patients with CRC [61]. Recent studies have identified many miRs in blood as prognostic or diagnostic biomarkers in CRC (Table 1 and Table 2).

## 4. Biomarkers for Prediction of Drug Efficacy

Drug resistance is a major obstacle to effective cancer therapy. Selecting patients who would benefit from drug treatment will help to promote therapeutic efficacy and avoid resource waste. miRs are expected to have applications as predictive biomarkers for therapeutic responses because some miRs have been shown to induce chemoresistance and to be associated with poor prognosis in various malignancies, including CRC [97,98,99,100].

Cohort research in the United States of America and China has shown that increases in *miR-21* expression are associated with resistance to 5-fluorouracil (5-FU) chemotherapy [22]. In this in vitro study, resistance was induced by inhibiting the DNA repair protein MutS homolog2 (MSH2). Additionally, *miR-140*, *miR-215*, *miR-224*, and *miR-20a* have also been reported to contribute to drug resistance. Currently, studies are underway to determine if the expression of these miRs can be used to predict chemotherapy efficacy or as treatment targets.

The list of miRs as predictive biomarkers of response to vascular endothelial growth factor- or epidermal growth factor receptor (EGFR)-targeted therapy and chemotherapy, which are used in the standard treatment regimen for CRC, is shown in Table 3.

## 5. Limitations

### 5.1. Conflicting Functions of miRs

Recently, studies have focused on the contrasting roles of miRs in oncogenesis and tumor suppression [38,116]. Some miRs have been reported to be upregulated in one report and downregulated in another report, indicating the contradictory functions of these miRs in CRC. For example, *miR-27a* was found to be downregulated and showed tumor-suppressive functions in CRC, targeting *Stat3* and *Smad2* [117]; in other studies [118,119], this same miR was found to be upregulated and showed oncogenic functions in CRC [120]. Additionally, *miR-155* has also been shown experimentally to have conflicting functions in mouse breast cancer [121].

Tumor-derived miRs may have site-dependent functions that promote tumor development. Thus, miR localization should be considered for clinical cancer screening or anticancer therapy for targeting miRs [63].

### 5.2. Sensitivity/Specificity

Despite the potential of individual miRs as biomarkers or therapeutic targets, combinations of miRs are expected to enhance the sensitivity and specificity for cancer diagnosis and increase the intensity of treatment [122]. Indeed, combinations of miRs have been shown to provide high diagnostic accuracy, with a high area under the curve, in lung [123], pancreatic [124], liver [125], and breast cancers [126,127].

Evaluation of miR panels using large, independent patient cohorts must be performed before miR biomarkers can be implemented in the clinical setting in CRC. Furthermore, combinations of some miR panels and cancer antigens or molecular biomarkers should be assessed as reported in breast cancer [127,128] and pancreatic cancer [124].

### 5.3. Internal Control

Endogenous control is crucial for the normalization, reliability, and reproducibility of diagnostic results because it helps to normalize differences among sample qualities and variations during the detection process. In pooled studies, internal controls have included *miR-16*, *U6*, and *miR-451*, of which *U6* and *miR-16* have been most popular [122]. However, it is still unclear whether these miRs are good internal references [129]. The suitability of the miR as a reference may depend on the type of organ or tissue. Caution is required when selecting an internal control gene for evaluating the expression profiles of miRs in patients with CRC.

## 6. Development of Novel Diagnostic Methods Using miRs

In Japan, the New Energy and Industrial Technology Development Organization (NEDO; http://www.nedo.go.jp/english/index.html) has carried out a collaborative industry/government/ academia project since 2014 to develop a cutting-edge, next-generation cancer diagnostic system (Project Number: 14009). Since 2015, this project has been now supported by the Japan Agency for Medical Research and Development (AMED). The objective is to construct a database of miR expression in body fluids, conduct a comprehensive analysis, discover early expression markers for 13 cancers (including breast cancer and CRC) or other diseases (such as dementia), and create biological tools that could be practically applied for the detection of these markers.

## 7. Conclusions

As highlighted in this review, miRs have considerable potential as biomarkers and therapeutic targets because miRs can drive and modulate tumorigenesis and tumor progression in CRC. However, the clinical significance of miRs as biomarkers is still not conclusive, and independent validation studies are needed for clinical application.

The use of miRs as biomarkers for CRC would provide a new, less-invasive technique to screen for CRC and determine prognosis. A screening panel consisting of multiple miRs may provide the most precise and effective screening tool for CRC.

Interestingly, recent studies have demonstrated that miRs may be critical regulators of immune responses, and aberrant expression or dysfunction of miRs in the immune system is associated with cancers [130,131,132]. Moreover, emerging evidence has demonstrated that immune-associated miRs are dysregulated in both tumor cells and immune cells, suggesting that miRs could be involved in communication between tumor cells and immune cells [133]. Much effort has been made to discover the precise role of miRs in the regulation of antitumor immune responses.

In this review, we found that most papers reporting the clinical significance of miRs in CRC were small-scale studies, and a meta-analysis was reported only for *miR-21* in CRC. More meta-analyses or large-scale analyses are required to provide reliable data for clarification of the clinical significance of miRs for clinical applications. A large prospective clinical study may be the best approach.

Finally, we hope that elucidation of the molecular mechanisms of miRs in CRC will lead to the discovery of early diagnostic methods or allow the development of next-generation oligonucleotide drugs and other novel therapies.

## Figures and Tables

**Table 1:** Tissue and blood microRNAs as prognostic biomarkers for CRC.

**Tissues**		
**microRNA**	Population with Poor Prognosis *	**References**
miR-7	up	[19]
miR-20a	up	[20,21]
	-	[22]
miR-21	up	[23], Meta-analysis: [24,25]
miR-22	down	[26]
miR-31	-	[15]
miR-92a	up	[27]
	-	[15]
miR-93	down	[19]
miR-101	-	[15]
miR-155	up	[28]
let-7c	down	[29]
miR-126	down	[30]
miR-130b	up	[31]
miR-132	down	[32]
miR-139-3p	down	[33]
miR-15	down	[34]
miR-16	down	[34]
miR-181b	up	[20]
	-	[22]
miR-183	up	[35]
miR-195	down	[19,36]
miR-196a	up	[37]
miR-196b	up	[37]
miR-203	up	[20]
	-	[22,38]
miR-215	up	[39]
miR-224	up	[40]
miR-340	down	[41]
miR-34-5p	down	[42]
miR-320e	up	[43]
miR-17-5p	up	[44]
miR-106a	down	[44]
	up	[20]
	-	[15]
miR-150	down	[45]
miR-29a	up	[46]
miR-372	up	[47]
miR-141	up	[19]
miR-144	down	[48]
miR-145	-	[15]
miR-181a	up	[49]
miR-494	up	[19]
**Blood/Serum/Plasma**		
**microRNA**	Population with Poor Prognosis *	**References**
miR-21	up	[50]
miR-96	up	[51]
miR-124-5p	down	[52]
miR-141	up	[53]
miR-155	up	[54]
miR-200b	up	[51]
miR-200c	up	[55]
miR-218	down	[56]
miR-221	up	[57]
miR-29a	up	[46]
miR-148a	down	[58]
miR-183	up	[59]
miR-345	up	[60]
miR-19a	up	[61]
miR-203	up	[62,63]

* up, upregulated expression in patients with poor prognosis compared with that in patients with good prognosis. down, downregulated expression in patients with poor prognosis compared with that in patients with good prognosis. -, no significant difference in prognosis between patients with upregulated and downregulated miRs.

**Table 2:** Blood and stool microRNAs as diagnostic biomarkers for CRC.

**Blood/Serum/Plasma**		
**microRNA**	Population with CRC *	**References**
miR-18a	up	[74,75]
miR-19b	up	[74]
miR-15a	up	[74]
miR-21	up	Meta-analysis: [24,76,77,78]
miR-24	down	[79]
miR-29a	up	[74,75,80]
miR-34a	down	[81]
miR-375	down	[82]
miR-409-3p	up	[83]
miR-7	down	[83]
miR-93	down	[83]
miR-17-3p	up	[84]
let-7g	up	[85]
miR-31	down	[85]
miR-181b	down	[85]
miR-203	down	[85]
miR-378	up	[86]
miR-20a	up	[75]
miR-143	up	[75]
miR-145	up	[75]
miR-133a	up	[75]
miR-106b	up	[75]
miR-335	up	[74]
miR-601	down	[87]
miR-223	up	[88,89]
miR-92	up	[75,80,84,88,89]
	down	[85]
miR-760	down	[87]
miR-423-5p	down	[79]
miR-320a	down	[79]
miR-19a	up	[74,78,88]
miR-425-5p	up	[78]
miR-422a	down	[88]
**Stool**		
**microRNA**	Population with CRC *	**References**
miR-17-92 cluster	up	[72]
miR-20a	up	[90]
miR-21	up	Meta-analysis: [76]
miR-135	up	[70,72]
miR-144 *	up	[91]
miR-29a	down	[71]
miR-223	up	[89]
miR-221	up	[73]
miR-92a	up	[69,89]
miR-224	down	[71]

* up, upregulated expression in population with CRC compared with healthy volunteers. down, downregulated expression in population with CRC compared with healthy volunteers.

**Table 3:** Tissue and blood microRNA biomarkers for therapeutic response in CRC.

**Tissues**			
**Therapy**	**microRNA**	Non-responders Pre-therapy *	**References**
Anti-angiogenetic therapy	miR-126	up	[101]
Anti-EGFR therapy	miR-7	down	[102]
Chemotherapy	miR-31-5p	up	[103]
	Let-7c	down	[104]
	miR-99a	down	[104]
	miR-125b	down	[104]
	miR-17-5p	up	[105]
	miR-21	up	[106]
	miR-143	down	[107]
	miR-148a	down	[108]
	miR-148b	down	[109]
	miR-150	down	[45]
	miR-200c	down	[110]
	miR-320	down	[111]
	miR-625-3p	up	[112]
	miR-181b	up	[112]
	miR-27b	up	[112]
	miR-664-3p	down	[113]
	miR-455-5p	up	[113]
	miR-196b-5p	down	[113]
	miR-592	down	[113]
**Blood/Serum/Plasma**			
**Therapy**	**microRNA**	Non-responders Pre-therapy *	**References**
Anti-angiogenetic therapy	miR-126	up	[114]
Anti-EGFR therapy	miR-345	up	[60]
Chemotherapy	miR-126	up	[114]
	miR-143	down	[107]
	miR-345	up	[60]
	miR-1914 *	down	[115]
	miR-1915	down	[115]

* up, upregulated expression at pre-therapy in non-responders compared with responders. down, downregulated expression at pre-therapy in non-responders compared with responders.
